# Sowing the seeds of transformative practice to actualize women’s rights to respectful maternity care: reflections from Kenya using the consolidated framework for implementation research

**DOI:** 10.1186/s12905-017-0425-8

**Published:** 2017-08-30

**Authors:** Charlotte E Warren, Charity Ndwiga, Pooja Sripad, Melissa Medich, Anne Njeru, Alice Maranga, George Odhiambo, Timothy Abuya

**Affiliations:** 1 Population Council, 4301 Connecticut Avenue NW, Suite, Washington, DC #280 USA; 2 Population Council, PO Box 17643-00500, Nairobi, Kenya; 3 Department of Family Medicine, UCLA David Geffen School of Medicine, 10880 Wilshire Blvd., Suite, Los Angeles, CA 1800 USA; 4 Division of Reproductive Health, Ministry of Health, PO Box 43319-00100, Nairobi, Kenya; 5 Federation of Women’s Lawyers, (FIDA) Kenya, PO Box 46324-00100, Nairobi, Kenya; 6 National Nurses Association: Midwives Chapter of Kenya, PO Box 49422-00100, Nairobi, Kenya

**Keywords:** Mistreatment, Respectful maternity care, Implementation, Disrespect and abuse

## Abstract

**Background:**

Despite years of growing concern about poor provider attitudes and women experiencing mistreatment during facility based childbirth, there are limited interventions that specifically focus on addressing these issues. The *Heshima* project is an evidence-based participatory implementation research study conducted in 13 facilities in Kenya. It engaged a range of community, facility, and policy stakeholders to address the causes of mistreatment during childbirth and promote respectful maternity care.

**Methods:**

We used the consolidated framework for implementation research (CFIR) as an analytical lens to describe a complex, multifaceted set of interventions through a reflexive and iterative process for triangulating qualitative data. Data from a broad range of project documents, reports, and interviews were collected at different time points during the implementation of *Heshima.* Assessment of in-depth interview data used NVivo (Version 10) and Atlas.ti software to inductively derive codes for themes at baseline, supplemental, and endline. Our purpose was to generate categories of themes for analysis found across the intervention design and implementation stages.

**Results:**

The implementation process, intervention characteristics, individual champions, and inner and outer settings influenced both *Heshima*’s successes and challenges at policy, facility, and community levels. Implementation success stemmed from readiness for change at multiple levels, constant communication between stakeholders, and perceived importance to communities. The relative advantage and adequacy of implementation of the Respectful Maternity Care (RMC) resource package was meaningful within Kenyan politics and health policy, given the timing and national promise to improve the quality of maternity care.

**Conclusion:**

We found the CFIR lens a promising and flexible one for understanding the complex interventions. Despite the relatively nascent stage of RMC implementation research, we feel this study is an important start to understanding a range of interventions that can begin to address issues of mistreatment in maternity care; replication of these activities is needed globally to better understand if the *Heshima* implementation process can be successful in different countries and regions.

## Background

Mistreatment of women during labor and delivery is a global challenge, because it negatively influences women’s decisions to seek future obstetric care at health facilities [1, 2] and violates women’s rights [3, 4]. Despite nearly two decades of growing concern about poor provider attitudes and women experiencing mistreatment in health facilities [2, 5–8], few maternal health service interventions have a central objective focusing on these issues. Instead they are embedded in interventions that focus on improving perceptions of quality of care [2, 9] or through subsidized consumer-led demand for maternal health services through the use of vouchers or demand side financing [10, 11]. Measuring mistreatment is difficult, it includes a variety of underlying and contributory factors such as normalized practices among providers in under-resourced facilities [5, 12, 13].

Disrespectful and abusive treatment has been defined as any interaction or facility condition deemed locally to be humiliating or undignified, as well as interactions or conditions experienced by women or intended to be humiliating or undignified by providers [14]. Manifestations include physical abuse, non-consensual, non-confidential, non-dignified care, abandonment or neglect, discrimination, and inappropriate demands for payment in health facilities [2]. The most appropriate and effective interventions for combating mistreatment have not been well documented. Aggravating and mitigating elements of poor provider-client relationships have been neglected in health systems research, particularly in maternity units [5, 8].

In 2010, the United States Agency for International Development (USAID)-funded TRAction Project commissioned a landscape analysis, on disrespect and abuse during facility based childbirth. This review by Bowser and Hill, provided the platform for addressing disrespect and abuse globally. At around the same time, the White Ribbon Alliance (WRA)– also supported by USAID – convened an advocacy group of policy makers, advocates, programmers, and researchers (the Respectful Maternity Care Advisory Council) which developed the Universal Rights of Childbearing Women [15]. The Advisory Council designated the term ‘Respectful Maternity Care’ (RMC) to promote interventions that mitigate the factors and effects of disrespect and abuse [15].

By 2011, concern for maternity healthcare had increased considerably in Kenya. Factors included the country’s high maternal mortality ratio (488 deaths per 100,000 live births), low proportions of facility deliveries (43%) [16], and a growing recognition that mistreatment was a barrier to maternity care. Moreover, a report documenting the issue co-authored by the Federation of Women Lawyers-Kenya (hereafter referred to as FIDA) [7], results from the 2010 Kenya Service Provision Assessment Survey [17], and media reports of poor quality of maternal health amplified the issue.

Concurrent with WRA’s advocacy agenda, the *Heshima* (“dignity” in Kiswahili) project in Kenya was tasked in 2011, by the TRAction Project, to determine and measure the prevalence of disrespect and abuse, conduct implementation research for developing and validating tools for assessment, to design interventions to address determinants of disrespect and abuse, and finally evaluate the effects of the interventions [18]. Building on the Bowser and Hill landscape analysis, the *Heshima* study incorporated policy-, facility-, and community-level perspectives in its design, implementation, and assessment [18, 19]. *Staha*, a sister project in Tanzania funded through TRAction with the same objectives [20], teamed with Heshima to review the manifestations of disrespect and abuse during childbirth and translate them into measurable domains, that harmonize and contextualize the working definitions of disrespect and abuse or mistreatment [14, 19].

*Heshima* was one of the first projects globally that measured the prevalence of disrespect and abuse during childbirth and designed and developed interventions based on the results from the baseline study. Evidence from the *Heshima* baseline survey showed that 20% of postnatal women (*n* = 641) interviewed after being discharged from 13 study sites across Kenya reported that they had felt humiliated at some point during labour and childbirth [19].

Given the dearth of literature and evidence in the field of RMC interventions, *Heshima* researchers and implementers followed an iterative and participatory process of learning-by-doing throughout all the phases of the project’s design, development, and assessment to build evidence and to implement a series of interventions that intersect policy, facility, and community levels. The intervention planned to analyze and address inequalities, discriminatory practices, and unjust power relations between providers and clients as defined by international human rights treaties and corresponding governmental statutes and laws [21]. Such an approach inherently prioritizes intervention acceptability and quality, as it draws upon layers of varying perspectives that recognize aspects of disrespect and abuse during childbirth in distinct ways [14].

The purpose of this paper is to capture and explain the complexity and interconnectedness of the elements of *Heshima* [22]. Using an adaptation of the consolidated framework for implementation research (CFIR) [23]- the paper both describes and analyses the implementation process, its strengths and challenges, and the lessons gained from the *Heshima* experience. In doing so, the paper emphasizes valuable aspects of its design that may be transferable to similar settings.

## Methodology

### Study context

*Heshima* is an evidence-based participatory implementation research study conducted in 13 facilities in five Central and Western Kenya counties that began in 2011. It engaged a range of community, facility, and policy stakeholders to address the causes of disrespect and abuse during childbirth and promote RMC [18]. The *Heshima* consortium was led by Population Council (hereafter known as The Council), an international research organization with an extended history (since 1960s) of operations research and support for policy and program development in Kenya with particular focus on quality of reproductive (and maternal) healthcare. The Council collaborated with FIDA, co-authors of *Failure to Deliver 2007*, which highlights issues of mistreatment, and advocates for women’s rights at local and national levels [7]. *Heshima’s* other key member was the National Nurses Association of Kenya/Midwifery Chapter (hereafter known as the Nurse/Midwife Association), a member of both the International Council of Nurses and International Confederation of Midwives, who empower their members (nurses and midwives) to provide quality care. The project steering committee included representatives of two departments within the Ministry of Health (MoH), the Division of Reproductive Health and the Department of Nursing; the Nursing Council of Kenya; and a core group of stakeholders interested in improving access to quality maternal and newborn health (MNH) care in a rights-based approach.

### Data sources

**Fig. 1:**
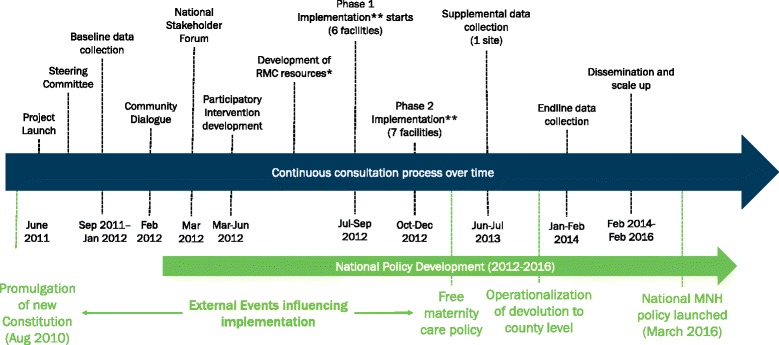
Heshima timeline of data collection and critical external factors

Data from a broad range of project documents, reports, and interviews were collected at different time points during the implementation of *Heshima*. A timeline (Fig. 1) depicts key events over the project period which guided research for this paper.

Continuous process documentation facilitated the triangulation of qualitative findings from focus group discussions, in-depth interviews, and dialogues with participants and beneficiaries. A detailed description of the methodology used for baseline and outcome data collection, is described elsewhere [18, 19, 24]. In brief, we conducted a before-and-after study designed to measure the effect of a package of interventions to reduce the prevalence of disrespect and abuse experienced by women during labor and delivery in 13 Kenyan health facilities. A range of empirical study tools were used (observations of client-provider interactions, client exit interviews, provider interviews, facility inventories). Prevalence data were collected through an exit survey of 641 women discharged from postnatal wards at baseline [13, 14], and compared with 728 at endline to assess the impact of the interventions. We also describe changes in observed behaviour at endline [18] (See project objectives in Table 1).

**Table 1:** Heshima project objectives

*Heshima* objectives:
1. Determine the manifestations, types and prevalence of disrespect and abuse in childbirth;
2. Develop and validate tools for assessing disrespect and abuse;
3. Identify and explore the potential drivers of disrespect and abuse;
4. Design, implement, monitor, and evaluate the impact of one or more interventions to reduce disrespect and abuse; and
5. Document and assess the dynamics of implementing interventions to reduce disrespect and abuse and generate lessons for replication at scale

Qualitative baseline and endline data were collected in September and October 2011 and January and February 2014, respectively, with a range of intervention participants. Supplementary data were retrieved from various sources such as summary reports, project reports and additional interviews. Summary reports were used from two critical meetings held in early 2012, a ‘community dialogue meeting’ and ‘stakeholder forum’, that disseminated baseline data to stakeholders and solicited recommendations for the development of a package of *Heshima* interventions. Information from these meetings was compiled and recorded in internal project reports. The complementary process documentation throughout the project period facilitated the translation of evidence into actions resulting in the consistent monitoring of intervention processes, notation of contextual effects, and afforded opportunities for addressing inherent challenges to implementation. Additional focus group discussions and in-depth interviews were obtained from a selected facility and its surrounding community between April and August, 2013 (Table 2).

**Table 2:** Data sources and study participants over the course of the intervention

Data Sources	Study groups	Timing and sample
In-Depth Interviews (IDIs)	District health program managers/coordinators	Baseline (*n* = 56)
	Health care providers (nurses, doctors)	
	Facility in-charges (nurse-midwives/matrons)	Supplemental data (*n* = 33)
	Community health workers	
	Traditional birth attendants	
	Policy makers (county-level)	Endline (*n* = 33)
	Professional associations (nursing, medical)	
	Women who delivered at a facility in the last 6 months	
Focus Group Discussions (FGDs)	Community members:	Baseline (*n* = 20)
	Single and multi-parity women (separate groups)	Supplemental
	Men in the community	data (*n* = 8)
	Opinion leaders (chiefs, elders)	Endline (*n* = 5)
Case Narratives	Women who delivered at a facility in the last 6 months	Baseline (*n* = 51)
		Endline (*n* = 14)
Process Documentation	Community dialogue report	Feb 2012
	National Stakeholder Forum report	March 2012
	Trip reports from comprehensive supervision visits	(*n* = 6)
	FIDA/ The Nurse/Midwife Association report(s) to	(*n* = 10 each)
	PC (quarterly). Steering committee meeting notes	(*n* = 12)
	Donor reports(quarterly /annual)	(*n* = 15)

Informed consent was obtained from all adult study participants. There were no minors included in the study*.* The research protocol was approved by the Division of Reproductive Health, Ministry of Health, as well as the Kenya Medical Research Institute (KEMRI)‘s Ethical Review Board (SCC 288) and the Council’s Institutional Review Board (Protocol 517).

### Analytical approach

This paper uses the CFIR as an analytical lens to describe a complex, multifaceted set of interventions through a reflexive and iterative process integral for triangulating qualitative data (23). CFIR is an amalgamation of several frameworks developed to evaluate complex intervention processes in the real world. It builds on theories of dissemination, innovation, organizational change, knowledge translation, implementation, and evidence-based interventions [23].

CFIR emphasizes stakeholder perceptions as central to the evaluation of an intervention from the design phase to intermediate and final outcomes by using five specific domains: intervention characteristics, inner setting, outer setting, characteristics of individuals involved, and process of implementation [23]. Currently, CFIR’s use has been limited to disease-specific or targeted behavior change interventions [25, 26]. We applied the analytic framework in an iterative process to describe the complexity of *Heshima’s* policy, facility, and community activities. The range of perceptions in *Heshima*’s qualitative evaluation, allowed us to deductively apply a modified version of CFIR (using a number of the constructs within the five domains) to gain an understanding of *Heshima*’s implementation process, strengths, and challenges. Thematic analysis by internal researchers (i.e. those directly involved in the implementation research) and external researchers (those with contextual knowledge) revealed gaps in addressing factors or drivers of disrespect and abuse at baseline (e. g. lack of awareness of rights for childbearing women, providers ‘carrying stress’), changes perceived by women delivering in study facilities, external influences experienced afterwards, and reflections about the process and outcomes of *Heshima* at endline (e.g. what worked well, or not, characteristics of individuals, influences on implementation).

Assessment of in-depth interview data used NVivo (Version 10) and Atlas.ti software to inductively derive codes for themes at baseline, supplemental, and endline. We generated categories of themes/issues for analysis found across the intervention design and implementation stages. Salient information extracted from process documentation (e.g. dates, coverage of activities, tools, outputs and outcomes) contextualized the implementation at the various intervention levels. Reflexive discussions of the data and broad issues faced during the implementation process included both internal (i.e. those directly involved in implementation) and external perspectives. This discursive, team-based methodology using CFIR corroborated multiple data sources, along thematically organized lines that fashioned inferences about *Heshima*’s implementation.

## Results

Findings are presented in two main sections that correspond with CFIR categorization: 1) the implementation process (intervention characteristics, individual characteristics and process domains) and 2) External and internal influences on intervention implementation (inner and outer settings and intervention contexts). Our findings are further disaggregated according to level of policy, facility, or community activities. Table 3 describes the different roles and influences of various institutions, participants and stakeholders (including the *Heshima* team) and differing degrees of influence during the intervention design implementation process.

**Table 3:** Roles and influence of different stakeholders

Stakeholders		Role	Level of Influence
Heshima Project Members	Population Council (The Council)	Led the consortium, designed the research, coordinated intervention. Long-term presence in Kenya (since 1960s). Respected by MOH (both as an institution - evidence for policy and individual staff). Engaged MOH prior to and during proposal development. Institutional knowledge existing maternal health research /evidence; Member of national technical working groups, support national policy, strategy and guidelines development. Rights-based approach to reproductive health services.	High
	Federation of Women’s Lawyers - FIDA	Promotes women’s rights through advocacy	Medium
		Build on work on meditation for inheritance, land disputes and documenting abuses to women.	
		Contributed to policy and community component	
	National Nurses Association of Kenya- Midwifery Chapter	Empower health providers to provide quality care	Medium
		Support health facility interventions: training in values clarification and attitude transformation, quality improvement teams, supervision with MOH	
Steering Committee	Kenya Obstetric/ Gynecological Society. University of Nairobi: Depts: Nursing, ObGyn. School of Public Health; WHO, UNICEF, MOH-Dept. of Nursing, Division of Reproductive Health, Nursing Council of Kenya	Policy level mechanism to provide feedback on study design and implementation process	Medium (as a group)
		Review /develop study instruments and design and monitoring of intervention	
MOH/public sector	MOH headquarters	Director of Public Health launched/supported project. Director of Medical Services committed throughout.	Medium
	Nursing Council of Kenya	Semi-autonomous institution - legislative responsibility for Nursing/ midwifery training curriculum and examining board. Introduced revised regulatory standards, scope of practice and ethical code in 2013	High
	Dept. of Nursing	Technical support /design of intervention/supervision	Medium
	Division of Reproductive Health	Technical support and policy guidance: participated in IR process, design and monitoring of intervention	High
	Department of Human Resources	Project results used to support health sector reforms to address drivers of mistreatment including provider accountability	Low
	County health management teams	Oversee policy implementation provide direction for implementation and monitoring result utilization.	Medium
	Facility/maternity unit managers	Oversee policy implementation and provide supportive environment for frontline providers and community	Medium –High
	Service providers: nurses doctors, midwives	Beneficiaries of training/Implement interventions	High
	Parliamentarians	Advocacy	Low
	Media	Advocacy	Medium
International NGOs	Reproductive health/ MNH partners: Jhpiego, IPAS, FCI, FHI360	Contributed to development of RMC resource package (training guide for facilitators, participants and communities) and advocacy.	Medium
Community	Community health extension workers	Linkage between facility and community. Oversee and train CHVs facilitate alternative dispute resolution	Medium
	Community health volunteers/Legal aid officers	Sensitize communities on universal rights, obligations for childbearing women and other rights issues.	Medium
		Sensitize how to demand for health rights and report incidents of mistreatment and help coordinate alternative resolution meetings	
	Community members	Perceptions of disrespect and abuse, awareness of rights at baseline and endline beneficiaries	Low
	Women	Perceptions of men extent of male involvement and how to support women	
	Men		
	Mothers who have delivered in facilities	Perceptions and experiences at baseline and endline	Low
Private sector/ Faith based Institutions	Civil society organizations CSOs:	Participate in policy discussions and dissemination	Low
	Private /faith based providers	Implementers of intervention	
	Reproductive Health Rights Alliance	Steering committee member	Low
Development partners	USAID /Washington and Kenya	Funded Heshima through TRAction Project (USAID/W) steering committee member (USAID Kenya)	Medium
	UNICEF - Kenya	Steering committee member	Low
	WHO –Kenya	Steering committee member	Low

### Implementation process

Table 4 provides a description of specific intervention activities at each level, including purpose, target group(s), coverage, and intensity at policy, health system and community levels; this includes the CFIR domains of individual and intervention characteristics, and the processes involved in the development, design, introduction and implementation at the three different levels.

**Table 4:** Heshima process description (including interventions)

	Intervention Activity	Purpose	Participants	Frequency	Duration	Location
Policy level						
1.	Project Launch	High level MOH officials launched Heshima. Media invited. Participants invited to Project Steering Committee to guide/monitor activities.	80	One time	One morning	Nairobi
2.	Project Steering Committee	Maternal health champions known to Heshima with mandate to support quality childbirth met routinely to review project progress	See Table 4	12	Half day	Nairobi
3.	Global Policy Review	Desk review of international conventions, treaties, signed by Kenya, national laws, and the new constitution for relevant policies and guidelines on human and childbearing rights for promoting and strategizing for RMC by The Council and FIDA.	FIDA lead	One time	20 days	Nairobi
4.	Baseline questionnaire development training of data collectors	Formative research conducted to understand the context and mistreatment terminology by communities and provider motivation and accountability issues prior baseline questionnaire development. Health managers from national nursing and reproductive health units were invited to be part of the data collector training. Specifically requested to coordinate observations of client provider interactions during labor and delivery in the 13 study facilities.	Heshima and steering committee Heshima (Council led), MOH, data collectors	Ad hoc meetings	30 days	Nairobi & non- study site
				One time	1 week	Nairobi
5.	Community Dialogue and National Stakeholder Forum	Community level findings disseminated in each facility catchment area Stakeholders: community, facility, national, global representatives. Baseline findings – drivers of disrespect and abuse (See Fig. 2) – disseminated then group work by level and county to suggest interventions to mitigate disrespect and abuse.	1996 community members 100 stakeholders	One time	1 day	Nairobi
6.	Participatory Intervention Development	Meetings with Heshima members and Steering Committee to review discussions from No.5.	20	Series of meetings	1 month	Nairobi
7.	Development of RMC Resources and Curricula	1) Baseline results and stakeholder consensus on the content of training materials; 2) Values components adapted from IPAS training materials; 3) Sessions on rights based approach, service charter including accountability. 4) Added professional code of ethics. 5) MOH convened national meetings on RMC curricula development for both pre- and in-service training; 6) Final face to face meeting with project partners and steering committee members refined the RMC resource package; and 7) Two international experts reviewed final version prior to completion. RMC components were incorporated into national curriculum.	10–15 national maternal health stakeholders 7 members Nursing Council of Kenya	3 meetings plus virtual experts in training material development	7 days 30 days	Nairobi
8.	National Policy Dialogue and Development	Policy engagement through the national reproductive health interagency coordination committee, technical working groups for maternal and newborn health, Human Resource and monitoring and evaluation. Presentations of results to get buy in prior to national dissemination. Meetings continued for scale up plans.	20–40 stakeholders /meeting	4–8 meetings (quarterly)	half day	Nairobi
		The Council participated in small expert meetings (invitees only) to draft Maternal Health Bill and ensure disrespect and abuse during facility based childbirth incorporated.	10–15 national stakeholders	15 meetings	Half day /meetings	Nairobi
		Working with the high level policy makers such as Kenya Women’s Parliamentary Association, the Parliamentary Health Committee and the first Lady to advocate for Reproductive /Maternal Health Rights.	Over 50	3 strategic meetings Plus ongoing	Half day meetings	Nairobi
9.	Advocacy	National conferences and meetings with media (e. g. Kenya Media Network), researchers, professional associations (midwifery, ObGyn and medical) and policy makers on health rights and promoting RMC. Continuous advocacy by MOH and Heshima.	120 national stakeholders	12 targeted meetings.	Half day national meetings	Nairobi / county level
Health system - Facility level						
10.	VCAT Workshops	RMC workshop (1–2 days) for county health managers, facility and maternity in charges, i.e. those who supervise /support frontline providers Three day workshop for facility staff. Each of the study facilities developed action plans to institutionalize RMC in maternity units.	132 146 Maternity providers	One time /county Facility reps to one meeting	2 -day workshop 3- days /workshop	All study Counties and facilities
11.	Mentorship	Following VCAT workshops, on-the-job role-modeling for provider behavior change by facility champions as part of routine continuous professional development.	13 identified, 4 actively engaged	Conducted as part of routine work	continuous on job session	4 sites: 2 public, 2 private
12.	Quality Improvements teams	Strengthened facility management and quality improvement teams to monitor, address, and resolve incidents of mistreatment. Address infrastructure, drugs and commodity supply concerns. Quality improvement teams trained on rights and obligations related to childbirth, developed protocol for reporting and monitoring, and encouraged community membership. Established mechanisms for transparency and accountability of health facilities to communities, increase awareness of maternal healthcare rights.	Public facilities −10; private facilities −0) 3–6 members	Quarterly review meetings 4 x year	Ongoing and 2–3 h meeting	All study counties
13	Counseling for providers	Counseling for providers at the group and/or individual levels to support providers with coping mechanisms to overcome experiences related to high workload, trauma or critical incidents. Conducted by FIDA counselors (one counseling session per site) and role modelled sessions with the facility or county counsellors. Counselors continued with counseling sessions in their respective sites.	113 providers (8–12/ site)	26 sessions; 9 sites one each. 4 sites; 3–4.	45 min- 1 h per session	All study counties
14	Maternity Open Days	Trust-building with local communities: men and women visit the facility to learn about procedures in the maternity wards and interact with staff.	100–300 depends on facility size	24 (total)	1 day each	All study counties
15	Monitoring of disrespect and abuse	Provided mechanisms to report incidents of disrespect and abuse such as customer service desks, suggestion boxes and through Heshima/MOH supervision. County health teams and facility quality improvement teams conducted monitoring and supervision as part of their routine work.	~350 community members ~35 at facilities	17 county visits 22 community health units; 13 facility visits**	½ day community health units and ½ day facility	All study counties
Community level						
16	Community workshops	One day workshop held for community resource persons (community health volunteers, legal aids, chiefs, religious leaders/village elders) on civic education of community rights to sexual and reproductive health including maternal health care. FIDA facilitated the workshop. CHEWs support community health volunteers to develop action plans.	154 community people trained	5 times (1 per county)	1 day each workshop	Catchment of all facilities
17.	Community education and male involvement	Community health volunteers, CHEWs, opinion leaders, civil and legal aids) conducted RMC sensitization meetings for community members with support from county mangers. Deliberate efforts were made to involve men in the community workshops as participants and facilitators. Targeted meetings for men: ‘calling them to action’ to demand RMC for their wives and partners.	1996 people: 287 male only, 871 female only, 838 mixed groups	27 meetings	Half day	Catchment areas from around facilities
18.	Mediation training for society leaders	Trained society leaders (e. g. CHVs), on mediation skills, to act as intermediaries between community members and health facility to address issues of disrespect and abuse. Mediators selected by communities and facilities (on set criteria) and trained by FIDA. *** Counseling community members who have experienced mistreatment. Led by FIDA and other professional counselors. Referrals from CHVs or community legal aids.	22 from community health units, 13 from facilities 2 out of 6 women	5 times (1 per county) Twice	1 day per session 1 h per session	All study counties One site

*FIDA uses routine lobbying processes on civic and women’s health issues to advocate of behalf of Heshima. **In some counties, community units shared between multiple facilities. *** In RMC Resource Package Manual

The immediate section below describes how *Heshima* gained high level support to then implement the project, followed by a description of the process and activities at the three different levels.

#### Project launch and project steering committee

In the early stages of the project (pre-research), rapport and ownership of *Heshima* was gained by continuous policy dialogue at technical meetings with government, civil society, and professional knowledge networks. Presence of and participation by *Heshima* staff compelled critical actors to reflect on RMC and disrespect and abuse as components of quality of maternity care. The project was launched by high level MoH representatives; key MNH actors the media were also invited. Project steering committee members were then selected from notable individual champions or institutions, based on their influence in MNH and rights-based approaches in Kenya (see Table 3). The steering committee convened quarterly to discuss findings, progress and resolve implementation challenges.

#### Global policy review and baseline survey development

To understand the broader policy context, FIDA conducted a review of international conventions, treaties, signed by Kenya, national laws, and the new constitution for relevant policies and guidelines on human and childbearing rights. This informed the formative research questions conducted prior to baseline questionnaire development to understand community and provider context and understanding of the issue. In order to provide a solid platform of engagement with MoH, health managers from the national nursing and reproductive health units were invited to be part of the data collector training. Specifically, they were requested to coordinate observations of client provider interactions during labor and delivery in study facilities. A senior health manager later reported, *“I have seen with my own eyes, disrespect and abuse is not necessarily due to lack of commodities or low staffing*,*”* and became one of *Heshima*’s greatest advocates.

#### National Stakeholders Forum and community dialogue

**Fig. 2:**
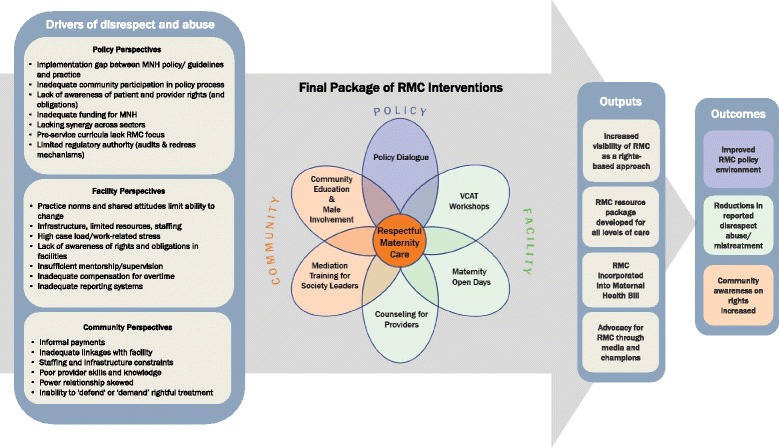
Heshima Theory of Change

*Heshima* shared preliminary analysis of baseline data (February 2012), with the steering committee and MoH and then held community dialogues at study facilities. *Heshima* convened a one-day stakeholder forum with over 100 participants: including community members from around study facilities, the steering committee, representatives from public, private and faith based study health facilities, national representatives from MoH, World Health Organization (WHO), United Nations Population Fund (UNFPA), United Nations Children’s Education Fund (UNICEF), academia, media, professional associations and international non- governmental organizations, global representatives from USAID and TRAction (see Table 3). The stakeholder forum disseminated community and facility baseline findings, which outlined the key drivers of disrespect and abuse shown in Fig. 2 at each level, built upon the recent community dialogues and local concerns about disrespect and abuse, and emphasized the need for incorporating RMC into a legal and policy framework. Participants discussed the findings and recommended intervention activities.

During the forum power dynamics were noted in mixed level discussions that were organized geographically. Sub-county health managers led discussions, while community members or frontline health workers made few remarks. This led to the commitment to establish regular ‘community dialogue’ sessions with local men and women as well as engagement with frontline providers to ensure their opinions were incorporated as the intervention evolved. Two health managers recognized issues that needed to be addressed to improve provider - client relationships:

*“The face of the hospital needs to change to build public trust”* (Nursing officer in-charge, hospital); and *“The staff morale needs to be boosted by a scheme of service. Satisfied care givers in turn make satisfied clients. It boosts the working relationship.”* (Senior officer, policy, MoH).

Some health managers felt threatened by the *Heshima* objectives, believing that providers were being targeted as offenders while a weak health system did not support good quality care; *“How do you expect a midwife to be in a good mood if she works with no break and has many clients to attend to in a dirty working environment?”* (Baseline health manager)*.*

#### Participatory intervention development

Deliberations from the stakeholder forum, *Heshima* partner experiences, and ongoing MoH and steering committee consultations resulted in a final package of interventions introduced in study facilities and their communities. The final package (described in greater detail later) includes:

1. Policy level: on-going advocacy and policy dialogue, including involvement in maternal health bill, and development of values clarification and attitude transformation (VCAT) training materials (in addition to the initial policy start-up activities described above).
2. Facility level: health manager and provider workshops on VCAT; counseling support for providers, strengthening quality improvement teams, strengthening community-health facility linkages, maternity unit open days for pregnant women and their families; and mechanisms for reporting and addressing disrespect and abuse.
3. Community level: interventions included community sensitization on the rights of childbearing women and mediation or alternative dispute resolution training (Fig. 2).

##### Policy level

In Kenya, there is a culture of stakeholder engagement in national policy making which provided opportunities for *Heshima*: “*The Kenya policy framework 2011-2030 is using a consultative process where all stakeholders are involved. For… specific policies like MCH, whatever is there, will be updated to fit what is being drawn for the whole sector”* (Baseline IDI, policy stakeholder). The Council and FIDA were notably influential in promoting RMC during smaller meetings with key political figures and national champions. Inclusion of RMC within policy or guideline arenas demonstrates policy success.

*Heshima* project was successful at influencing policy. Policy activities promoted attitude change, advocacy, and improved awareness in study counties, nationally and internationally by demanding continuous engagement of stakeholders with different levels of influence. The Nursing Council of Kenya incorporated RMC training into the national nurses’ curriculum. RMC language and goals were also incorporated into the technical content of the Maternal, Newborn and Child Health Bill that arose from continued involvement of *Heshima* members in informing and writing policy. Another success of *Heshima’s* consultative process is the multi-stakeholder involvement in the development of the RMC resource package (Table 4). This includes an adaptation of VCAT training originally developed by IPAS [27] to empower private practitioner provision of post-abortion care, provides a means for adapting the reflexive concept for factors influencing disrespect and abuse, and integrates broader professional ethics, rights, and accountability updates.

##### Health system interventions

ᅟ

#### Values clarification and attitude transformation workshops

Sub-county and facility managers attended a one-two day workshop to warrant their understanding of the RMC concept and all other components of the intervention. This was followed by a three-day RMC workshop for providers who were offered an opportunity to identify factors they wished to address (team or individual) with the help of standard self-reflection and teamwork tools for continued self-assessment and improvement. Both workshops targeted improving attitudes, working environments, facility capabilities, and community links for accountability and governance. Sessions were structured on defining the drivers of disrespect and abuse (See Fig. 2) and RMC, by emphasizing international and national laws and conventions, treaties on reproductive health and human rights, professional ethics, and facility management. Sessions also addressed providers’ and clients’ rights and obligations during childbirth and provided methods for critical self-evaluation of individual behaviors and attitudes that might contribute to disrespect and abuse.

Despite sub-county health managers plans to include RMC updates beyond the initial workshops in continuous professional development seminars, the sessions tended to focus more on clinical skills than on the ‘soft’ RMC issues. However, providers’ understanding of health as a right and mitigating disrespect and abuse improved. It also enhanced providers’ self-awareness of norms, attitudes, and behaviors during service delivery with some success. *“It has taught me how to handle the patient with care, to respect their rights and to follow what we were taught in college that we should not abuse patients… Heshima taught us the right way to treat the patient”* (Endline, case narrative with nurse manager, public facility); and “*Our capacity has really been built to promote dignified care—looking back we are surprised at how we treated mothers then*” (County health manager, 2013).

#### Mentorship

The RMC package includes a mentorship approach to promote and improve quality of care in labor and delivery services. RMC workplans designate responsible staff in maternity units to share their skills and foster peer knowledge about mistreatment and RMC. This promotes and enriches discussions on these issues and improved team and individual accountability: *“I would say attitude changed—attitude, attitude, attitude—the attitude of the workers has really changed. It is not business as usual anymore and it is very positive”* (Endline, provider, hospital).

Principal challenges regarding mentorship during *Heshima* were staff shortages and heavy workloads. Mentors and mentees had little to no time with one other due to heavy workloads or the reassignment of mentors to other departments. Mentorship was more successful in four public facilities, where selected mentors were intrinsically committed to the *Heshima* cause (individual champions) and remained in their post for the duration of the intervention.

#### Quality improvement teams and reporting mechanisms for disrespect and abuse

The Kenya MoH mandates quality improvement teams for all health facilities. *Heshima* worked with these teams, strengthened them where necessary, and ensured maternity staff were included in the quality improvement teams. The Nurse/Midwife Association also supported these teams by reviewing duty rosters and ensuring appropriate staff allocation and deployment despite low staffing levels. Other changes implemented included posting documents that explained client rights and obligations and service charters in maternity wards, installing suggestion boxes, conducting exit interviews for quality assurance, and establishing public relations personnel desks in maternity units. Despite an overall enhancement of resource management at endline (supplies and maternity drugs), essential commodities remain insufficient and inconsistent.

Common obstacles preventing regular team meeting included time constraints, competing tasks, and travel expenses for community members. Following the RMC workshops, providers developed individual and team action plans. Some facility teams offered each other peer support and received feedback from each other on observable behaviors such as verbal abuse, abandonment. These were discussed in staff meetings (e.g. shift changeover meetings). In some facilities staff complaints were also addressed in monthly or ad hoc facility meetings. In addition, once quality improvement teams formed their RMC plans, some facilities began to reward the good performance of midwives with a range of low cost awards including photos, certificates, or medals to incentivize further improvement.

Mechanisms for reporting disrespect and abuse (suggestion boxes, exit interviews, client reports) were instituted by these teams and had some positive effect. *“We have a customer care point where customers can give either their compliments or complaints. We have several suggestion boxes where customers can anonymously communicate with us. We have advisory committees that now handle cases which are felt that maybe need to be interrogated further or some disciplinary cases which are to be reviewed”* (Endline IDI, health facility manager, public facility). However not all suggestions boxes were used and community engagement was preferred for giving feedback.

#### Counseling for health providers in maternity units

This centered on providers’ well-being through the provision of psycho-social support focusing on managing workloads and challenges to promote RMC. FIDA and the Nurse/Midwife Association, with health managers’ support, offered both group and individual counseling sessions (for those requiring extra sessions) to all providers. Stress management support was highly appreciated, easily adapted and institutionalized into existing facility and referral structures: “*All providers in maternity unit reported that the session helped them off load the ‘baggage’ they have been carrying”* (Endline, nurse manager, public facility). Some providers preferred outside counseling, indicating a need for confidentiality and privacy. Smaller facilities accessed services from larger facilities through day visits. Although providers and other staff trained on counseling (nurse/midwives, psychologists, and chaplains) were available they were underutilized, which has implications for sustainability.

#### Maternity open days (Fig. 2)

This component invites surrounding communities into maternity units. It attracts both community and provider support, improves relationships, helps dispel myths and misconceptions associated with facility delivery, and forms good community relationships. *“I have seen… there are no ropes to tie one to the delivery bed during childbirth”* (Primigravida, experience of Maternity Open Day in a public facility, 2013); and “*We have included this in our annual work plan and will be funded by the government since we have established such a good relationship with the community that we want to continue”* (Endline, IDI, health manager, public facility).

Other service demonstrations were incorporated into Maternity Open Days (Table 4), to encourage participation, including health talks, child welfare clinics, and screenings for other conditions (such as cervical and prostate cancer). Maternity Open Days improved the awareness of management committees of the needs and functions of their maternity units, as well as demonstrating a marked increase in male participation (Table 4). The concept followed similar processes at all the intervention sites and, despite some logistical challenges, maintained successful implementation.

#### Monitoring

*Heshima* conducted regular meetings, follow up, and dialogue in all study counties and facilities (Table 4) who received three or four monitoring visits overall. Around 80% of providers from the study maternity units were assisted with identifying mechanisms for sustaining positive attitude change. While most health managers recognized and endorsed the need for RMC, observations during monitoring visits consistently found that the subject of disrespect and abuse (and motivations for addressing it) elicited a variety of reactions including denial and defensiveness, or at least justification for the behavior. “*That issue of the health worker feeling that they are being targeted and the clients are not also being targeted to also change was probably a challenge… maybe the health worker is feeling unfairly as the target that they are always the cause of abuse”* (Endline IDI, health facility manager, public facility). However, interviews with women who had recently given birth in health facilities revealed positive changes among providers. *“You see that harassment, shouting at people, slapping people, all that is no more… In 2007, when I came to deliver my firstborn son, they never used to talk to people nicely. But now they talk to you like your sister or mother, very nicely… So you don’t even fear going there”* (Endline, case narrative, public facility).

##### Community interventions

Community level interventions were led by FIDA with support from The Council (Table 3). There were varied degrees of engagement and execution at this level.

#### Community workshops

Working with facility health management teams, community health extension workers (CHEWs) and other community individuals, 30 male and female community health volunteers were trained as community RMC trainers using the RMC resource package [28]. *“For me, I learned that education is key. Since after we were trained we trained others and we have seen many changes due to this” (*Endline*,* focus group discussion, community health volunteer*).*

Others noted that *“There are a lot of lessons like communication… it is now at all levels where you are communicating horizontally, downward and even to your seniors during advocacy—I have leant it is a very important skill. I have also learnt the health system itself has a lot of insufficiencies”* (Endline, in-depth interview, community health volunteer).

#### Community education and male involvement

Community RMC trainers initiated discussions during community dialogue sessions about the treatment of women typically experience during childbirth. Next, explicit education on health rights, particularly those pertinent to RMC [15], was followed by a question and answer session to guarantee group comprehension. Community RMC trainers also emphasized male involvement to reduce disrespect and abuse; including emphasis on the need for birth companionship (male partner or female family member). Community members were given printed information on RMC, legal mechanisms available, and how to report occurrences of disrespect and abuse. Women who had experienced any mistreatment were offered counseling.

#### Mediation/alternative dispute resolution

FIDA led this activity – training community personnel in the mediation process as a mechanism to resolve incidents of mistreatment. Mediation uses an independent and impartial third party who facilitates the negotiation process that brings aggrieved parties together providing an opportunity for solutions that may be locally acceptable. Mediation supports both the providers’ and clients’ rights and obligations by ensuring each side is heard [28]. Despite community enthusiasm for mediation activities, FIDA received few referrals for counseling or legal actions, and some sites received no reports of mistreatment at all. The underlying fear of public accusation of facilities inhibits mediation was apparent throughout.

“*They [*women*] still have that fear of reporting because you can get someone complaining but when you ask for their name they are still afraid”* (endline, community health volunteer).

Facilities interested in protecting their image limited mediation activities. In some circumstances community members wanted more than the actual redress, however in three facilities community members clearly stated that a simple apology from management and providers allowed for amicable resolution. “*We discussed the case, the midwife apologized for her mistakes… and the hospital management took her for a course on public relations to learn more on customer care... before this we rarely discussed cases of rudeness”* (Endline, nurse-in-charge, maternity unit, public hospital).

### External and internal influences on intervention implementation

This section builds on the CFIR’s three other domains (inner, outer and intervention contexts) in relation to the implementation process. Damschroder describes implementation as a constellation of processes; and how the social processes are intertwined within the context of where it takes place [23, 29]. The outer or external influences described below include the free maternity policy, actors monitoring activities and networks and communication. Internal influences are described as part of community relations and quality improvement and facility-community –centric influences, and the final CFIR domain (individual characteristics influence) of the intervention process.

#### Free maternity policy

A free maternity policy was instituted in Kenya, following a Presidential decree in 2013 and contributed in drawing maternity care to the forefront of public attention. Concurrent with the latter stages of *Heshima’s* implementation the free maternity care decree affected the intervention at all levels. Despite the policy’s positive intention, communities remained skeptical about how free maternity affects quality of care, particularly with the increased demand on health facilities, delayed financing from central level, augmented provider workloads and shortages.

*“So unlike in the initial stage of Heshima project when nurses would take time to listen to patients, nowadays the patients are just too many yet the nurses are very few… yes it is true* Heshima *has done a good job and the mothers are being treated well but the problem is that nurses are experiencing many challenges now”* (Endline, focus group discussion, community health volunteer).

#### External monitors

The role of external actors (*Heshima* implementation team and steering committee) in monitoring the intervention was essential for providing outside perspectives that balanced the layers of perceptions for necessary changes affecting disrespect and abuse. An external monitoring body can be internalized as long as the community plays a significant role in providing recommendations. Following the initial workshops, community RMC Trainers were encouraged to record their activities and discuss progress through *Heshima* monitoring visits and community dialogue days. Using a participatory approach to monitor progress can enhance community buy-in to the extent that activities become the norm and therefore sustained –as indicated below:

*“I think there’s need for enhanced monitoring and evaluation...You know, if we have sustained midway whereby you support us, and we do it, you also come to verify what, if actually we are doing is what you want us to do…I would urge that we be engaged fully and there be kind of checks and balances … which will assist the program to be sustained, so that after some time it can even be done without Heshima in the picture”* (Endline, focus group, community health volunteer).

#### Communication

The intervention did improve communication between health facility management teams and maternity staff, as well as between facilities and communities. However, this was highly contingent on both ‘inner’ and ‘outer setting’ influences and/or challenges. The free maternity mandate posed challenges to the ‘outer setting’, by influencing the form and function of the facility management as a result of a reduction of discretionary funds available from cost sharing sources. Before, maternity services funds were used to purchase out of stock items and were not included in routine expenditures.

#### Community relations and quality improvement teams

Some health managers demonstrated positive working relations with community members in dealing with disrespect and abuse, while others remained indifferent to community involvement despite improved relations between frontline providers and community members. This indifference posed potential obstacles in some sites for the quality improvement teams’ capacity to strengthen facility and community links. The propagation of ‘fear of facilities’ may have limited the extent to which complaints were acknowledged: “*Perhaps they fear us. That training gave us a lot of power to question the service providers if they have done wrong*” (community health volunteer, supervision visit, 2013). Variations emerged for how facilities institutionalized disrespect and abuse reporting: Some resolved cases amicably and informally, while others were left unsettled. One community health volunteer reported his frustration at not being able to negotiate a mediation session for a disrespect and abuse report and being told by a health manager, *“We are looking into the staff issue first, that is our responsibility and we know we have a problem as managers we want to deal with this”* (community health volunteer, supervision visit).

#### Facility and community-centric influences

Centralized management structures in private or faith-based facilities were resistant to change; some staff feared losing their jobs. For instance, some health managers and providers expressed concern that media coverage would propagate fear of facilities and perceptions that providers are not performing their duties. This in turn had implications for monitoring progress and disclosure to consortium partners. For maternity open days, some sites only permitted women (not men) to enter their maternity ward, citing lack of privacy and poor infrastructure, while others only supported partial openness (e.g. permitted RMC promotional videos, but no ward visits). Despite these challenges, facilities implemented this component favorably (Table 3).

Community-based workshop on childbirth disrespect and abuse identification and reporting, health rights, legal and maternity procedures, and mediation sessions were replicated successfully, with high coverage (Table 3). However, community trainers sometimes faced external challenges such as weak community units and insufficient funding for community work. Trainers were challenged by the complexity of their role as educators, mediators, monitors and reporters of disrespect and abuse to FIDA or facilities. Of the six cases of disrespect and abuse reported during community dialogues privately after community workshops, only two cases were relayed to county health management teams.

#### Individual characteristics influencing implementation process

Some providers, who felt isolated as ‘perpetrators’ of disrespect and abuse and were sole change agents (as opposed to clients) may resist implementation. Similar patterns may be seen among providers expecting extrinsic motivators (compensation) for their attitude and behavior change.

“*She* [provider] *was thinking there will be some* [monetary] *motivation from somewhere so that they are able to implement… that was a challenge… I told them motivation is from within so they should not be expecting any motivation from outside”.* (Endline, health manager, public facility).

Although the Nurse/Midwife Association and FIDA had their own ‘mandate’ within the project, their influence and reach varied across study sites. In some cases, representatives from the Nurse/Midwife Association were also members of facility management teams, signifying contradictory roles that tempered implementation effect.

Some community level trainers spoke of a need for longer initial workshop and frequent follow up mechanisms with *Heshima*, particularly when encountering community reluctance. *“It was a bit difficult for us to actually grasp everything… We were given manuals to use… to train. But when we came out to the field, and started meeting different groups…* [dialogue participants] *didn’t want to speak out because they said we were spying for* [X facility]*”* (Endline, IDI, CHEW).

## Discussion

### Utility of CFIR

The CFIR framework is a useful organizational tool for contextually understanding and adapting interventions to country needs, as in the *Heshima* case, noting barriers to implementation (staff turnover, resistance to change) and facilitators (staff continuation, champions, supervisors, local employment). In applying CFIR to triangulate a range of qualitative data, we considered inner and outer settings integral to the implementation process and useful for reviewing context, which affected stakeholder participation at the design, development, and implementation stages. Although the scope of study did not allow for application of all CFIR components, as the analytic guide was considered retrospectively after data collection, it served as a valuable framing tool. Application of CFIR broadly to policy, facility, and community activities suggests that its categories—intervention, inner setting, outer setting, characteristics of individuals, and process—provide moderate utility in discussing a complex, multifaceted intervention. However, some of the more detailed domains (28 in all) in the CFIR were not used as they were not contextually relevant. Given this is one of the first studies to apply CFIR in sub-Saharan Africa, we recommend further use and testing of the framework to different multifaceted interventions and health areas in the region.

This paper shows that a participatory, iterative approach is critical for designing, planning, and executing a complex, multifaceted package to mitigate factors of disrespect and abuse. All levels were affected by shifting policy and readiness for change within facilities and communities, as well as cultural beliefs and practices and characteristics of individual champions. This implementation research sought to improve knowledge of disrespect and abuse, and health as a human right, for both clients and providers; improve self-awareness of how socio-norms, values, attitudes, and behavior affect service delivery during labor and childbirth; support individual and facility cultivation of professionalism and mutual respect, among providers, clients, and communities; improve individual and team accountability; and devise mechanisms for dealing with drivers of mistreatment.

The implementation process is an inter-related series of sub processes that do not necessarily occur sequentially. Successful implementation often requires an active change process [23]. Implementation success, evidenced in Kenya, is rooted in readiness for change at multiple levels, constant communication between stakeholders and implementing partners, as well as perceived importance and benefits to communities. The relative advantage and adequacy of implementation of the RMC package was meaningful within Kenyan politics and health policy, which exhibited readiness for quality improvement in maternity care. Shifting health infrastructure affected *Heshima* implementation, and clarifications of roles and links among the various levels of activities will help strengthen implementation in the future. The consultative process is at the heart of implementation success, in a multifaceted approach addressing RMC. The flexibility to accommodate the changing policy environment (both local and national health strategies) allows its transfer to other contexts.

### Readiness for change and continuous engagement

In Kenya, intense public interest in maternity care and health rights were generated by media coverage of RMC efforts, the participatory design of *Heshima* interventions, and free maternity care. National scale up requires sustained commitment and formation of new partnerships with local organizations. Global acceptance—an indirect consequence—is most vividly seen in the WHO statement calling attention to the promotion of dignity and access to maternity care [30]. The WHO’s statement was influenced by *Heshima* and the RMC global partnerships through their participation in global technical working groups, advisory consultations, and international conferences. Despite early emergence of disrespect and abuse as an issue in South Africa, Ghana [5, 8, 31, 32] and Kenya, noted by FIDA and the Centre for Reproductive Rights [7], little response was elicited. The Bowser and Hill landscape analysis was followed by calls for proposals, high level donor engagement, WRA advocacy role, and the *Heshima* and *Staha* projects, resulting in sustained global interest. Since 2010, many MNH strategies and policies include RMC. Continuous engagement and discussion are congruent with the current Kenyan policy process, but implementation of policy activities met considerable challenges, including limited political and financial resources during the election campaigns of 2012–2013 and the ambiguity of oversight roles and funds dispersion in the devolved government system. Some political aspects posed challenges to implementation (e.g. free maternity), while others created windows of opportunity (outer setting) for raising awareness of RMC, such as discussions with high profile individuals (champions) and a readiness (inner setting) to change at policy, facility, and community levels [23].

Readiness for change is critical to ensure implementation of the maternity unit activities. Maternity unit activities confronted challenges similar to other studies in Africa, including inadequate knowledge and skills combined with broader health systems failures and low staffing [33, 34]. Prior attention to macro issues related to workforce training, recruitment, retention and distribution recently shifted to human resources for health for strengthened health systems [35]. Focus has turned to health workers capabilities, their motivations and other structural and organizational aspects of systems that influence and moderate workforce performance including leadership and supervision (mid-level management) and communication [34–36].

#### Flexibility and adaptability

Despite RMC’s moderately standardized training, the flexibility of the participatory process remained fundamental to the approach. Acceptance of the issue, both nationally and locally, was also exemplified by iterations of the *Heshima* RMC strategy, which combines the lessons on replicating a process of developing and implementing an adaptable, multifaceted intervention approach in Kenya or similar settings. Other factors, such as facility proximity, transportation, ability to pay, social norms, family decisions, and culture, contributed to the outcomes [7, 37]. Social norms, for example, reveal that men have roles to play, as protectors and household decision makers. Since men often hold leadership positions in their communities, their involvement is critical in catalyzing changes in facilities that lead to RMC.

#### Inner and outer settings: Implementation challenges

### Shifting policy structure

In 2010 Kenya promulgated a new Constitution which was subsequently inaugurated in 2013. The critical political impact on the health system (and all other governmental administration) was devolution from national/central control to 48 counties. This posed complexities for financing and governance while the new county health teams worked out their roles and management processes in the devolution transition in 2013. The free maternity care mandate introduced by the new president (2013) revealed a political interest in increasing access to care but created, and continues to create, health system and human resource challenges at county level. The devolution of the health sector and free maternity policy resulted in two health workers strikes about job security and left providers and managers uncertain about providing maternity services without secondary resources, thus negatively impacting the project’s continuity as well as the quality of care. Reporting challenges experienced by facilities and communities possibly result from a broader health sector re-organizations that affected the administrative roles of county officials and their relationships with facility management teams. Those shifting relationships posed uncertainty about RMC’s importance in the implementation of the MNH guidelines and reproductive health policy [38, 39].

### Facility and provider support

As much as there were improvements, facility infrastructure issues including limited space, lack of equipment and supplies, and staff shortages persist, that inhibit providers from offering quality care. Communities were frustrated by lack (in some facilities) of openness and commitment from health managers. Some managers continued to deny that disrespect and abuse existed and became defensive when incidents were reported. Some institutions professing support for RMC had individuals on staff not in concert with their organizational ethos. Even at endline a few health managers were not convinced disrespect was a legitimate issue or that allowing communities more say was ‘appropriate’. Regardless of reductions in provider rudeness and increased community willingness to anonymously report complaints, the reluctance to publicly report remains.

A key component welcomed by frontline providers was the recognition of their need for support. Just inviting them to discuss their work environments and challenges was an achievement that boosted their morale. Providers’ group counseling or stress management sessions with external counselors helped but were challenged by external factors such as delays in mutually agreeable dates for both counselors and maternity units. External counselors were a primary component of the study’s success, as most frontline providers were wary of disclosing issues in front of their managers, but after initial sessions most facilities were able to identify internal counselors, including hospital chaplains.

The quality of relationships and networks between *Heshima* partners, community health volunteers, opinion leaders, facility providers, and management affected community targeted implementation. The inherently political process of empowering communities, lodging complaints, and creating a systems environment conducive to criticism or mediation were challenges for implementing this level of intervention. Monitoring and evaluation however was not only critical to the iterative process of developing and conducting formalized *Heshima* RMC interventions, but they also may potentially sustain (to an extent) the facility and community dialogue after funding expires.

### Willingness to report and discuss disrespect and abuse

One major challenge in communities was the reluctance of women and their families’, to fully report incidents of mistreatment. Men, women, and community health workers were all empowered to ‘complain’ after learning their rights, but many were unwilling to act as witnesses and go through the mediation process, when they would have to face their perpetrators, fearing retribution from providers on their next visit. Many individuals felt certain they would be treated improperly should they need a particular provider’s help in the future. Health managers did not want to discuss their staff shortcomings with the community. While any mediation process is challenging, and although mediation and alternative dispute resolution training was seen as useful, for this to effectively ameliorate the impact of disrespect and abuse, more time, structured processes are required for communities and health managers are to understand its effective use. Moreover, as seen from community and facility perspectives, utilizing a collective accountability strengthening approach may offer a more feasible and effective strategy than case-by-case mediation.

### Broad scope of project

Some facility and community responses indicated *Heshima* may not have provide as much support as they wanted or needed. The project was ambitious in introducing a diverse intervention package, in a rapidly changing policy environment. However, introducing the intervention to 13 different facility types (hospital, health center, public, private, faith-based) in culturally different urban and rural areas provided the MOH and policy makers with evidence and promising practices that can work in a range of settings and gives credence to the adoption of RMC within national guidelines (launched in 2016).

### Strength of partnership and forging new ones

Institutional knowledge of key *Heshima* and steering committee members and an understanding of the political and policy environments were critical. Kenya’s political environment was very receptive after the adoption of the populist 2010 Constitution, in which a rights-based approach to health was clearly delineated. *Heshima*’s relative advantage*,* was its advocacy base and political support in Kenya across all intervention levels. Accommodating multiple perspectives was particularly important for promoting ownership of RMC interventions and resonated within the broader rights-based discourse in the region [7, 40, 41]. *Heshima’s* uniqueness likely influenced its implementation, and may not have been so successful were the consortium not so entrenched in Kenya, having strong relationships with MOH (including people at different levels), policy institutions, and partners, and using a rights-based approach. General openness to critique by the *Heshima* team provided an environment for frank dialogue on understanding this complex issue.

## Recommendations

### Participatory and consultative process is essential

Engaging stakeholders, nationally and locally, to agree that disrespect and abuse is an issue that needs to be addressed ensures the acceptance of an intervention. The focus of the consultative process should be an investment by direct, committed implementers (policy champions, facility contacts improving care quality, community health volunteers), including key coordinators (consortium partners) who serve as critical liaisons in a complex, multifaceted venture.

### Respectful maternity care workshops

Adding RMC to routine emergency obstetric and neonatal care skills training has been suggested, but while skills are essential for providing quality care, the ‘soft issue’ of respect for both clients and providers alike is not something to be appended to another workshop. Provider ‘attitude’ has been discussed frequently for decades, but nothing has changed. Until providers feel valued themselves, and work in a positive, enabling environment, these challenges will remain. We recommend that RMC workshops are held as standalone entities through professional development avenues and involve the whole maternity unit as a team. However, it is critical that counseling services, mentoring and good management structures are in place to support frontline providers in maternity units.

### Community involvement

Facilities that made the most progress were those that had supportive managers, proactive health workers who embraced building linkages with community members. Maternity open days were extremely successful in initiating the dialogue. Community sensitization regarding both their rights (and obligations) helped raise awareness of the challenges providers face and how communities can support health facility management*.*

### Consensus building is essential

The nature of RMC discussions during the *Heshima* experience varied between forums, groups of doctors, midwives, researchers, advocates, health managers, funders, and others. Regardless the compelling issue of addressing disrespect and abuse, built a consensus for the need of a multifaceted approach that resonated from all perspectives. Contextualizing interventions to mitigate disrespect and abuse in particular countries or in particular locales must take into account facility organizational cultures and community norms regarding health facility use.

## Conclusion

We found the CFIR overarching structure supports the exploration of essential factors to understand such a complex intervention. As one of the first global RMC implementation research efforts, we feel this study is an important start to understanding a range of interventions that can begin to address issues of mistreatment in maternity care. Replication of these activities is needed globally to better understand if the *Heshima* implementation process can be successful in different countries and regions.

## Abbreviations

CFIR: Consolidated framework for implementation research
CHEW: Community health extension worker
FIDA: Federation of women’s lawyers
KEMRI: Kenya medical research institute
MNH: Maternal and newborn health
MoH: Ministry of health
RMC: Respectful maternity care
TRAction: Translating research to action (project)
UNFPA: United Nation's Population Fund
UNICEF: United Nations Children’s Education Fund
USAID: United States Agency for International Development
VCAT: Values clarification and attitude transformation
WHO: World Health Organization
WRA: White Ribbon Alliance
